# Could type 2 diabetes be a component of the post-intensive care syndrome?

**DOI:** 10.1186/s13054-017-1607-3

**Published:** 2017-02-08

**Authors:** Jean-Charles Preiser, Caroline de Longueville

**Affiliations:** Department of Intensive Care, Erasme University Hospital, Université Libre de Bruxelles, 808 route de Lennik, 1070 Brussels, Belgium

The current definition of the post-intensive care syndrome (PICS) includes new or worsening impairments in physical, cognitive and/or mental health [1]. This list can encompass a broad number of dysfunctions, syndromes or specific diseases. In addition to muscular weakness and post-trauma stress disorder cognitive, behavioral or mental changes, other disorders may arise as a result of the pathophysiological events of the acute phase of critical illness, including physiological changes or the effects of therapies.

We here suggest that type 2 diabetes mellitus can be acquired during a stay in an intensive care unit (ICU-acquired diabetes (ICU-AD)) as an additional component of PICS. ICU-AD will increase the risks of long-term complications and increase the burden of critical illness. Regarding the pathogenesis of ICU-AD, persisting insulin resistance (IR) is the most likely candidate because IR develops rapidly during the acute phase of critical illness, with stress hyperglycemia (SHG) representing its commonest expression [2–4].

In contrast to the hyperglycemia in type 2 diabetes, which results from the combination of progressive IR developing over years and eventually culminates in beta-cell secretory defect, the pathogenesis of SHG involves a much quicker and more dramatic interplay of counter-regulatory hormones such as catecholamines, growth hormone, cortisol and cytokine, resulting in IR and raised hepatic glucose production (from gluconeogenesis and glycogenolysis) [3]. The adaptive mechanisms developed to survive a stress (sympathetic activity, release of pituitary hormones, inflammatory mediators) all result in a resistance to anabolic signals, including insulin [2, 3]. Teleologically, IR is considered an evolutionary preserved mechanism designed to supply higher amounts of glucose to insulin-independent tissues than to insulin-dependent tissues [2]. In the presence of IR, the flow of glucose will rise during the acute post-injury phase, triggering SHG [4]. The magnitude of IR should be confirmed by accurate assessments using isoglycemic hyperinsulinemic clamps. Because this technique can hardly be used in daily clinical practice, the use of physiological modeling including the determination of insulin sensitivity can be very helpful and may help to improve the quality of glycemic control.

In addition to IR, other stress-related pathophysiological derangements can also contribute to the development of ICU-AD. Among other potential mechanisms, a partial inhibition of the incretin effect could be involved. Such inhibition results in a lesser release of insulin in response to enterohormones released by the duodenum, small intestine or colon in response to oral or enteral food ingestion than in healthy conditions. Finally, a role of adipokines in the modulation of IR is suspected but not convincingly supported.

By analogy with gestational diabetes, a normalization of the sensitivity to insulin is expected after the resolution of the underlying condition. Contrasting with this belief, cohort studies have described the acquisition of type 2 diabetes, sometimes diagnosed very late after the resolution of the critical illness. Of note, the British National Institute for Health and Clinical Excellence [5] already recommended in 2011 screening for diabetes in patients who developed SHG after an acute coronary syndrome.

Abdelhamid et al. [6] reported recently in *Critical Care* the results of a systematic review of the incidence of diabetes of prediabetes diagnosed at least 3 months after hospital discharge of critically ill patients. Overall, this meta-analysis supports SHG as a risk factor for incident diabetes, when the risk of subsequent diabetes in patients who experienced SHG was multiplied by 3.5 [6].

Even though these data are quite convincing, the incidence of ICU-AD might be overestimated in some studies because diabetes might be preexisting in some patients [7, 8]. Taken together, these findings underline the relevance of a systematic determination of HbA1c on admission to diagnose unknown diabetes, or to assess the quality of glucose control in patients with known diabetes. Alternatively, the determination of the optimal BG target might be calculated from the value of HbA1c, as a potential strategy during a stay in the ICU [9, 10].

Regardless of the underlying mechanism, the acquisition of type 2 diabetes suggested by the results of reverse epidemiology techniques used by Abdelhamid et al. [6] is a major discovery, and represents a strong incentive for prospective studies to assess the actual incidence of ICU-AD and related risk factors. Indeed, in spite of the consistency of the data reported, there are several divergent aspects that cannot be assessed from cohort data. First, the definition of “new-onset” SHG varies from one study to another. Ideally, the absence of preexisting diabetes should be checked by the medical history and glycated hemoglobin below 6.5%, which is not available in most studies. Second, the severity and duration of SHG should be reported using standard metrics and completed by therapeutic data; that is, the amount of insulin required to achieve the target glucose level. The determination of a hyperglycemic index (area under the curve above the upper limit of the target blood glucose range) could be helpful in this regard. Likewise, the doses of some confounding medications such as steroids or catecholamines and the physical activity of the patients should be recorded to tease out changes in IR and hyperglycemia. Third, the diagnosis of diabetes should rely on the current definition based on an oral glucose tolerance test and/or the level of glycated hemoglobin. Fourth, the duration of follow-up should be considered carefully, because the timing for diagnosis of diabetes or prediabetes differs widely between studies. These divergent aspects should be carefully assessed when designing prospective collections in follow-up studies.

In conclusion, we urge for a thorough follow-up of patients after discharge from the ICU including screening for ICU-AD. An early diagnosis of ICU-AD will allow the early institution of appropriate treatment and the identification of risk factors will help to prevent it. If neglected, ICU-AD would represent a major public healthcare issue, as do the other components of PICS.
